# Effect of different transverse reinforcement confinements on circular columns cast with recycled concrete aggregates and treated wastewater

**DOI:** 10.1038/s41598-025-29810-9

**Published:** 2025-12-10

**Authors:** Abdallah M. Elmasry, Mohamed K. Ismail, Ahmed M. Farahat, Ahmed A. Elansary

**Affiliations:** Department of Structural Engineering, Faculty of Engineering, Cairo University, Giza, Egypt

**Keywords:** Reinforced concrete circular columns, Recycled coarse aggregate, Treated wastewater, Uniaxial axial compression, Transverse reinforcement confinement, Engineering, Environmental sciences, Materials science

## Abstract

The use of recycled concrete aggregate (RCA) and treated wastewater (TWW) in the concrete industry promotes sustainability, conserves potable water, reduces environmental impact, and minimizes waste, offering an eco-friendly alternative to conventional concrete production. However, RCA and TWW can negatively impact concrete performance, as RCA’s high variability, porosity, adhered mortar, and crushing-induced defects weaken the microstructure, while TWW’s dissolved salts and organic compounds interfere with cement hydration. Therefore, it is essential to fully understand their effects on concrete performance at both the material and structural levels and develop strategies to mitigate these drawbacks. Contributing to this, the present study investigated the effect of using RCA and/or TWW on the strength of concrete and the behavior of reinforced concrete (RC) circular columns. A total of 10 mixes were developed with RCA replacement levels of 0%, 30%, 50%, and 100%, and TWW replacement levels of 0%, 50%, and 100%. These concrete mixes were used to cast 24 RC columns, which were tested under monotonic concentric load. The columns were reinforced with different types of transverse reinforcement—stirrups at various spacings (200 mm, 150 mm, and 100 mm) and spiral reinforcement at different pitches (60 mm and 40 mm)—to provide varying levels of confinement, aiming to mitigate the negative effects of RCA and TWW on column capacity. The results indicated that the capacity of columns negatively affected by incorporating RCA and/or TWW. In columns with 200 mm spaced stirrups, as the RCA replacement increased from 0 to 30% and 50%, the failure loads decreased by 16.1% and 29.8%, respectively. The use of closely spaced stirrups or spiral reinforcement provided higher confinement, effectively alleviating the reduction in capacity caused by the use of RCA. Reducing the stirrups space to 100 mm allowed for up to 30% RCA replacement with no reduction in capacity, and up to 100% RCA replacement with a maximum capacity reduction of around 14.9%. Similarly, the use of spiral reinforcement at pitch of 40 mm enabled the use of up to 100% RCA with a reduction of 8.6%, compared to corresponding columns cast with no RCA. Higher reductions in capacity were observed when TWW was used, which further limits its application in concrete.

## Introduction

Rapid global growth in population and infrastructure has intensified the demand for concrete, the construction industry’s most widely used material. Although valued for its strength, durability, and versatility, large-scale concrete production heavily relies on natural resources such as natural coarse aggregates (NCA) and potable water (PW), raising concerns about their long-term sustainability. Simultaneously, this growth generates substantial concrete waste from renovation, reconstruction, and infrastructure expansion activities^1–3^. If unmanaged, this waste contributes to environmental pollution and landfill pressure but also offers opportunities for resource recovery. Through processing and crushing, concrete waste can be converted into recycled concrete aggregates (RCA)^4–10^, which can partially or fully replace natural aggregates in new concrete. This approach reduces demand for virgin resources and minimizes waste disposal impacts, promoting a more sustainable and circular construction industry.

Water, as a vital and limited resource, must also be used judiciously. The construction industry consumes large quantities of PW, highlighting the need for alternative water sources. Treated wastewater (TWW) can serve as a promising substitute, particularly in water-scarce regions^11^. However, the use of RCA and TWW requires careful evaluation due to potential impacts on concrete durability, strength, and overall performance. RCA often exhibits variability in size, shape, and composition because it is derived from heterogeneous construction and demolition waste, which can affect mix consistency and quality. Similarly, TWW may contain dissolved salts, sulfates, and chlorides that disrupt cement hydration, producing weaker hydration products and a less dense matrix. It can also carry organic matter and residual chemicals that retard setting and weaken the bond between cement paste and aggregates. Additionally, suspended solids and fine particulates can alter the effective water-to-cement ratio, increase pore connectivity, and elevate porosity, further compromising strength and durability^12–14^. Consequently, understanding these effects is crucial for optimizing mix design and implementing strategies to mitigate potential drawbacks.

Several studies have extensively examined the impact of using RCA on the properties of both plain and reinforced concrete (RC). In the case of plain concrete, research consistently showed that replacing natural aggregates with RCA generally leads to reductions in compressive strength, tensile strength, workability, and durability due to the higher porosity and weaker interfacial transition zones associated with recycled materials^2,15–20^. Surendar et al.^17^ found that increasing RCA content up to 75% led to reductions in compressive and splitting tensile strengths by 25% and 34%, respectively. In this study, a 10% replacement level was recommended as optimal, providing the highest strength with minimal water absorption. In another study, Abera^18^, reported that compressive strength decreased with increasing RCA content, although replacement levels up to 35% caused only minimal reductions. Bhat^19^, explored the influence of the original concrete’s quality—from which RCA was derived—on the mechanical properties of the resulting concrete and found that the source concrete’s grade had no significant effect, regardless of RCA content. On the other hand, Zong et al.^20^, in their study reported no strength reduction with RCA. This was because RCA’s higher water absorption lowered the effective water-to-cement ratio, offsetting potential strength losses from multiple interfacial transition zones (ITZs).

In terms of structural behavior, researchers have also explored the use of RCA in RC columns. For example, Ajdukiewicz and Kliszczewicz^21^ conducted an experimental study comparing rectangular RC columns made with RCA or NCA. The results showed similar bearing capacities, but columns with RCA exhibited significantly greater deformations. Choi and Yun ^22^, found that RAC columns generally exhibit similar cracking patterns and axial behavior to NCA columns, with a maximum capacity reduction of approximately 6–8% when 100% RCA is used. Moreover, the axial load capacity of columns with RCA met the ACI 318^23^ design strength criteria. Shatarat et al.^24^, also investigated the axial compressive behavior of 15 square RC columns using four types of aggregates: NCA, recycled asphalt pavement (RAP), RA, and RAP-RA. The results indicated that For RAP columns, axial load decreased by 2.95–31.06% and for RCA columns by 5.87% (RCA20) to 28.27% (RCA100) compared to NCA. Maximum recommended replacement levels are 20% for RCA-only, RAP-only, and combined RAP-RCA, and ACI 318^23^ and Japanese^25^ code equations remain applicable for yielding conservative estimations. Similarly, Pradhan et al.^26^, studied square columns with 100% RCA under eccentric axial loading, using two tie spacings (200 mm and 155 mm). Columns with RCA showed an 18% reduction in axial load capacity compared to NCA columns at the same transverse reinforcement ratio. Reducing tie spacing increased axial load capacity by 2.7% for NCA columns and 2.8% for RCA columns. Other contradictory results^27,28^ showed that using RCA can sometimes yield higher load-carrying capacity than columns cast with NCA.

Regarding TWW, Asadollahfardi et al.^29^ studied using TWW before chlorination to produce and cure concrete. Compared to control specimens, the tensile and compressive strengths of the TWW specimens were 96 ~ 100% and 93 ~ 96%, respectively. Meena and Luhar^11^, examined the mechanical and durability properties of concrete using tertiary TWW (TTWW), secondary TWW (STWW), and PW. For concrete with 100% TTWW, compressive strength decreased by 14.3% when cured with PW and 20.6% when cured with TTWW. Concrete with 100% STWW showed reductions of 28.0% and 29.2% for PW and STWW curing, respectively. Flexural strength was also lower than the control, decreasing by 8–20% for TTWW and 30–45% for STWW concrete. Raza et al.^30^, studied the influence of wastewater taken from domestic sewage (DSW), fertilizer factory (FFW), textile factory (TFW), sugar factory (SFW), and service station (SSW) on the mechanical properties of RCA plain concrete. It was reported that the compressive strengths of mixes cast with TFW, FFW, SSW, SFW, and DSW were 119%, 91%, 96%, 90%, and 51%, respectively, compared to those made with potable water. All wastewater types were high in organic matter (BOD5 and COD) and total suspended solids, except TFW, explaining why concrete made with TFW had greater strengths than those made with other wastewater types. Abushanab and Alnahhal^31^, also showed the use of TWW reduced concrete compressive and flexural strengths by 5 ~ 12% while it increased porosity and chloride permeability by about 40%. TWW also enhanced the workability of fresh concrete. Ahmed et al.^32^, examined the properties of concrete with TWW and RCA using a replacement ratio of 20% and cured using different types of water (PW, TWW, and 6%NaCl salt water (SW)). The results showed that long-term exposure to TWW and SW decreased the splitting tensile strength by 23.6% and 18.2%, respectively. Using 20% RCA and TWW had little effect on concrete compressive strength with TWW or seawater curing but resulted in a significant 22% reduction under PW curing. Abushanab and Alnahhal^33^, also studied the effects of using TWW and RCA, fully replacing natural gabbro aggregate and potable water, respectively. TWW reduced compressive and flexural strengths by 6–12% but lowered chloride permeability by 77% compared to PW concrete, while RCA decreased compressive and flexural strengths by 21% and 10%, respectively, compared to NCA concrete. Morgado et al.^34^, investigated the influence of TWW at replacement levels of 0%, 50%, and 100% on the mechanical properties of concrete. The compressive strength of concrete made with NCA and 50% or 100% TWW showed only slight reductions of 3% and 2.4%, respectively, compared with NCA concrete prepared with PW.

To mitigate the drawbacks of using RCA and TWW while maximizing their use in concrete, research efforts have explored various treatment and strengthening techniques^35–39^. Contributing to this direction and to the advancement of sustainable construction materials, this study first investigated the individual and combined effects of RCA and TWW on the structural behavior of RC circular columns. RCA was used as a partial or full replacement for NCA at replacement levels of 0%, 30%, 50%, and 100%, while TWW was used as a substitute for potable water (PW) at 0%, 50%, and 100% replacement levels. In addition, the role of transverse reinforcement was evaluated by employing both stirrups and spirals—stirrups were spaced at 200 mm, 150 mm, and 100 mm, and spirals were provided at pitches of 60 mm and 40 mm. The effectiveness of these reinforcement types in providing confinement and compensating for potential reductions in strength and ductility caused by RCA and TWW was assessed. Column behavior was examined in terms of crack patterns, ultimate load capacity, and load–displacement response. Furthermore, the accuracy of existing design codes in predicting the axial capacity of RC columns incorporating RCA and/or TWW was evaluated. The findings contribute to the optimization of RC column design using sustainable materials, ensuring structural performance is not compromised.

## Research significance

This study supports sustainable construction by investigating the use of RCA and TWW as eco-friendly alternatives in concrete production. Using RCA reduces reliance on natural aggregates and diverts construction waste from landfills, while TWW helps conserve PW—both critical for reducing the environmental footprint of the concrete industry. Unlike available studies, this research firstly investigated different replacement levels of RCA and the use of TWW, both individually and in combination, highlighting their effects on the performance of concrete circular columns. Secondly, it aimed to present practical solutions for using these waste materials while maintaining adequate structural capacity. To achieve this, the study focused on transverse reinforcement detailing, rather than other strengthening techniques, and demonstrated how confinement provided by various types and arrangements of transverse reinforcement could compensate for potential strength reductions. By assessing the structural performance of reinforced concrete columns made with these materials, and exploring reinforcement strategies to offset strength loss, the study offers practical solutions for integrating sustainability without compromising safety. The findings contribute to greener construction practices and informed material selection in structural design.

## Experimental program

### Materials

**Fig. 1:**
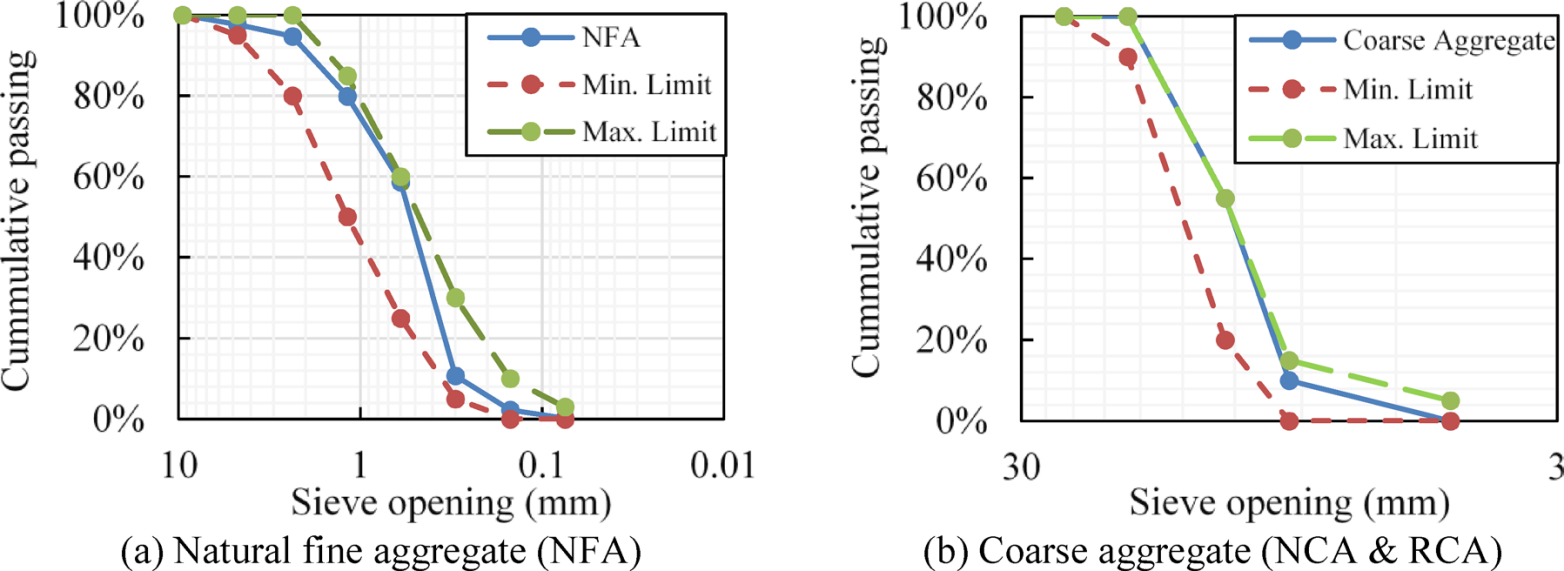
Sieve analysis for natural and recycled aggregate.

A total of ten mixes were developed in this study. Ordinary Portland cement type I was used as the binder in all mixes, while natural sand served as the fine aggregate. Two types of coarse aggregates were used: (a) NCA: gravel with a maximum size of 19 mm, and (b) RCA, made by crushing waste from tested standard cubes (150 × 150 × 150 mm) with compressive strengths ranging from 25 to 30 MPa, and sieved to obtain grading similar to NCA. The gradation curves for all coarse and fine aggregates used are shown in Fig. 1, and Table 1 lists their measured physical and mechanical properties. The water used was of two types: (a) PW and (b) TWW sourced from a domestic TWW plant in Cairo, Egypt, employing a treatment process based on activated sludge methods. The PW and the TWW satisfy the ASTM C1602^40^ and the ECP 203^41^ requirements, as shown in Tables 2 and 3, except for the difference in the initial setting time between TWW and PW slightly exceeds the ECP 203^41^ limit.

**Table 1:** Physical properties of natural and recycled aggregate.

Type of aggregate	Specific gravity (S.G) (ASTM C127^42^		Wet (bulk) unit weight (kg/m^3^) (ASTM C29^43^)	Water content (ASTM C566^44^)	Absorption (ASTM C127^45^)	Crushing index (BS 812–110^46^)**
	Oven dry (OD)	Saturated surface dry (SSD)				
NCA	2.50	2.55	1550	0.67%	1.67%	4%
RCA	2.54	2.67	1468	2.75%	4.80%	15%
FA	2.58*	2.63*	1670	0.95%	2.0%*	–

Standard test method is ASTM C128^47^. **The maximum aggregate crushing value is 30% (BS 812–110^46^).

**Table 2:** Chemical compositions of PW and TWW compared to available standards.

Element	PW (mg/l)	TWW (mg/l)	ASTM C1602^40^ (mg/l)	ECP 203^41^ (mg/l)
Chlorides as (${\text{Cl}}^{-}$)	34	121	< 1000	< 500
Sulfates	49	71	< 3000	< 1000
Total dissolved (TDS)	203	497	< 50,000	< 2000
P.H	8.02	7.81	–	7–9
Alkali carbonate and bicarbonate	15	119	< 600	< 500
Sodium sulfide	10	25	–	< 100
Organic	Nil	Nil	–	< 200
Inorganic (clay, suspension)	Nil	2.5	–	< 3000

**Table 3:** Setting time of cement paste.

Element	PW (minutes)	TWW (minutes)	Difference between TWW and PW (%)	ASTM C1602^40^ (deviation from control)	ECP 203^41^ limits
Initial setting time	120	155	+ 29.2	− 60 to + 90 min	Min. 45 min & difference ≤ 25%
Final setting time	250	215	− 14	− 60 to + 90 min	Max. 720 min & difference ≤ 25%

For columns reinforcement, deformed steel bars with a nominal yield stress of 350 MPa and a diameter of 10 mm were used for longitudinal reinforcement, while smooth steel bars with an 8 mm diameter and a nominal yield stress of 350 MPa were used for transverse reinforcement. The mechanical properties of the steel bars are provided in Table 4.

**Table 4:** Mechanical properties of steel bars.

Bar diameter (mm)	Elongation (%)	Yield stress, f_y_ (MPa)	Ultimate stress, f_u_ (MPa)	Elastic modulus, E_s_ (GPa)
10	17	321	461	204.8
8	20	311	455	186.9

### Concrete mixes

The ten concrete mixes developed in this study were detailed as follows (see Table 5):

- The first mix, designated as 0W0, was developed as a normal concrete mix using NCA and PW, serving as the control mix.
- The second, third, and fourth mixes, designated as 3W0, 5W0, 10W0, were developed similarly to 0W0, but with NCA replaced by RCA at replacement levels of 30%, 50%, and 100%, respectively. These mixes aimed to assess the effect of blended coarse aggregates to the full use of RCA on the behavior of RC columns. The RCA replacement levels were selected to examine how limited-to-moderate partial replacement (30% and 50%) and full replacement (100%) of NCA affect concrete performance.
- The fifth and sixth mixes, designated as 0W5 and 0W10, were developed similarly to 0W0, but with PW partially (50%) and fully (100%) replaced by TWW. These mixes were designed to evaluate the feasibility of using TWW as an alternative to PW in concrete production for structural applications. The TWW replacement levels were chosen to evaluate the impact of partially (50%) and fully (100%) replacing PW on concrete performance.
- The seventh to tenth mixes, designated as 3W5, 3W10, 5W5, and 5W10, were developed by simultaneously replacing NCA with RCA and PW with TWW at specific levels: 30% or 50% replacement of NCA with RCA, and 50% or 100% replacement of PW with TWW.. These mixes were designed to investigate the interaction between RCA and TWW in producing environmentally eco-friendly concrete suitable for structural applications.

**Table 5:** Mixture proportions of concrete.

Mix	RCA%	TWW%	PW (kg/m^3^)	TWW (kg/m^3^)	Cement (kg/m^3^)	Sand (kg/m^3^)	NCA (kg/m^3^)	RCA (kg/m^3^)
0W0	0	0	218	0	400	691.5	999.8	0.0
3W0	30	0	218	0	400	691.5	699.9	314.1
5W0	50	0	218	0	400	691.5	499.9	523.4
10W0	100	0	218	0	400	691.5	0.0	1046.8
0W5	0	50	109	109	400	691.5	999.8	0.0
0W10	0	100	0	218	400	691.5	999.8	0.0
3W5	30	50	109	109	400	691.5	699.9	314.1
3W10	30	100	0	218	400	691.5	699.9	314.1
5W5	50	50	109	109	400	691.5	499.9	523.4
5W10	50	100	0	218	400	691.5	499.9	523.4

It should be noted that the proportions of each mix were determined by systematically replacing a portion of the original materials—NCA with RCA and PW with TWW—while keeping the remaining mix components (cement and water) the same as the control mix (0W0). The sand content was slightly adjusted to account for differences in specific gravity between NCA and RCA.

In all mixes, the designation refers to the levels of aggregate and water replacement. For example, 3W5 indicates that 30% of NCA was replaced by RCA, while 50% of PW was replaced by TWW.

### Test specimens

**Fig. 2:**
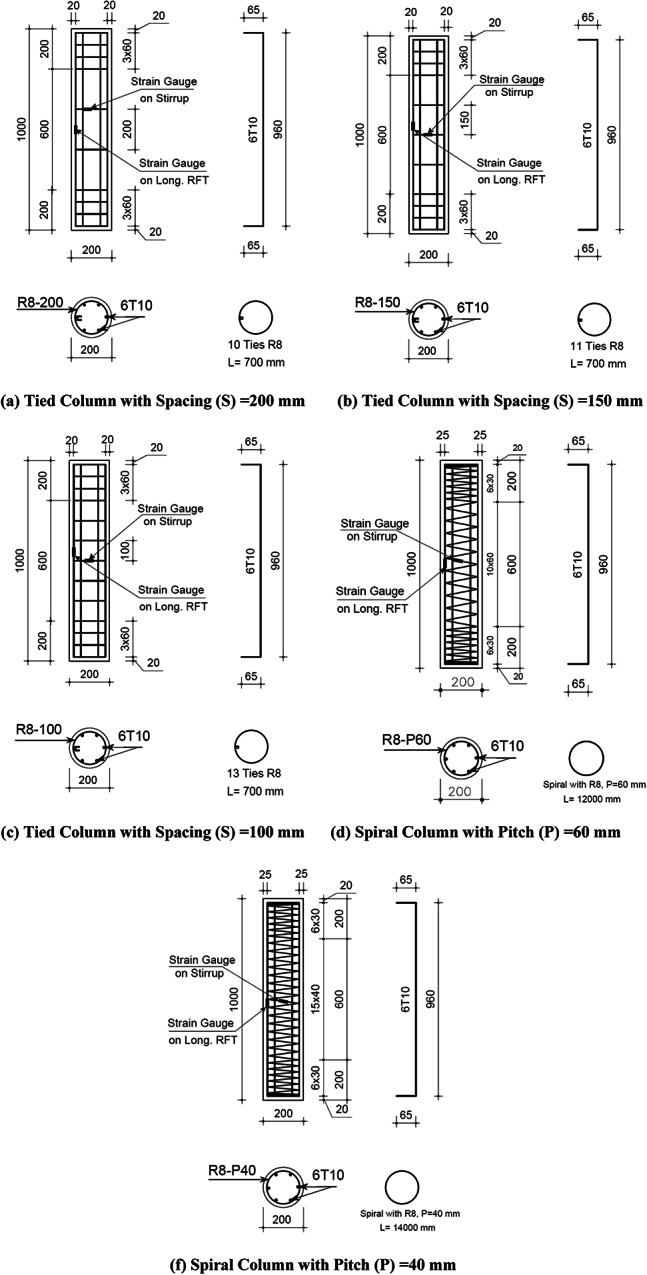
Dimensions and reinforcement details of tested columns.

In total, twenty-four circular short columns, each with a diameter of 200 mm and a height of 1000 mm, were cast in this study. All column specimens were longitudinally reinforced with six 10 mm diameter bars and transversally reinforced with either spirals or circular stirrups, having varied spacing or pitch, as shown in Fig. 2. Both circular stirrups and spiral reinforcement had a diameter of 8 mm. The longitudinal and transverse reinforcements met the minimum and maximum reinforcement requirements outlined in the ACI 318^48^ code. The twenty-four columns (C1–C24) were detailed as follows (see Table 6):

- Columns C1 to C3 were cast with mix 0W0 (control mix) and reinforced with circular stirrups at spacings of 200 mm, 150 mm, and 100 mm, respectively. These specimens were used to evaluate the effect of different confinement levels on normal concrete and also served as control specimens for stirrups-reinforced columns with mixes incorporating RCA and/or TWW.
- Columns C4 to C10 were cast with mixes 3W0 or 5W0 or 10W0 and reinforced with circular stirrups at spacings of 200 mm, 150 mm, and 100 mm. These specimens were designed to assess the effect of different confinement levels using circular stirrups to counteract the negative impacts of incorporating RCA at 30%, 50%, and 100% replacement levels. It is worth noting that with 100% RCA replacement, the optimal stirrups spacing (100 mm) was used.
- Columns C11 and C12 were cast with mix 0W0 (control mix) and spirally reinforced at pitches of 60 mm and 40 mm, respectively. These served as control specimens for spirally reinforced columns with mixes incorporating RCA and/or TWW.
- Columns C13 and C18 were cast with mixes 3W0 or 5W0 or 10W0 and spirally reinforced at pitches of 60 mm and 40 mm, respectively. These specimens were designed to evaluate the impact of various confinement levels using spiral reinforcement to mitigate the negative effects of RCA incorporation at 30%, 50%, and 100% replacement levels.
- Columns C19 to C24 were cast with mix 0W5, 0W10, 3W5, 3W10, 5W5, and 5W10 with circular stirrups at spacing of 200 mm. These specimens were designed to assess the effect of incorporating different combinations of RCA and TWW compared to 0W0 (control mix).

**Table 6:** Designation, replacement ratios, and lateral reinforcement details of tested columns.

Serial	Designation	RCA%	TWWR%	Type	Spacing/pitch (cm)	Stirrups volumetric ratio%
C1	0W0S20	0	0	Tied	20	0.4
C2	0W0S15	0	0	Tied	15	0.54
C3	0W0S10	0	0	Tied	10	0.8
C4	3W0S20	30	0	Tied	20	0.4
C5	3W0S15	30	0	Tied	15	0.54
C6	3W0S10	30	0	Tied	10	0.8
C7	5W0S20	50	0	Tied	20	0.4
C8	5W0S15	50	0	Tied	15	0.54
C9	5W0S10	50	0	Tied	10	0.8
C10	10W0S10	100	0	Tied	10	0.8
C11	0W0P6	0	0	Spiral	6	2.23
C12	0W0P4	0	0	Spiral	4	3.35
C13	3W0P6	30	0	Spiral	6	2.23
C14	3W0P4	30	0	Spiral	4	3.35
C15	5W0P6	50	0	Spiral	6	2.23
C16	5W0P4	50	0	Spiral	4	3.35
C17	10W0P6	100	0	Spiral	6	2.23
C18	10W0P4	100	0	Spiral	4	3.35
C19	0W5S20	0	50	Tied	20	0.4
C20	0W10S20	0	100	Tied	20	0.4
C21	3W5S20	30	50	Tied	20	0.4
C22	3W10S20	30	100	Tied	20	0.4
C23	5W5S20	50	50	Tied	20	0.4
C24	5W10S20	50	100	Tied	20	0.4

The tested columns were designated as either αWβSγ or αWβPγ for specimens with ties or spiral lateral reinforcement, respectively. In these designation, α refers to the RCA replacement level (0, 3, 5, or 10 corresponding to 0%, 30%, 50%, and 100%, respectively); W stands for TWW; β indicates the percentage of TWW used in mixing (0, 5, or 10 corresponding to 0%, 50%, and 100%, respectively); Sγ refers to the tie spacing in columns transversally reinforced with circular stirrups (20, 15, or 10 cm); and Pγ represents the spiral pitch in columns transversally reinforced with spirals (6 or 4 cm).

### Fabrication, casting, and curing of specimens

**Fig. 3:**
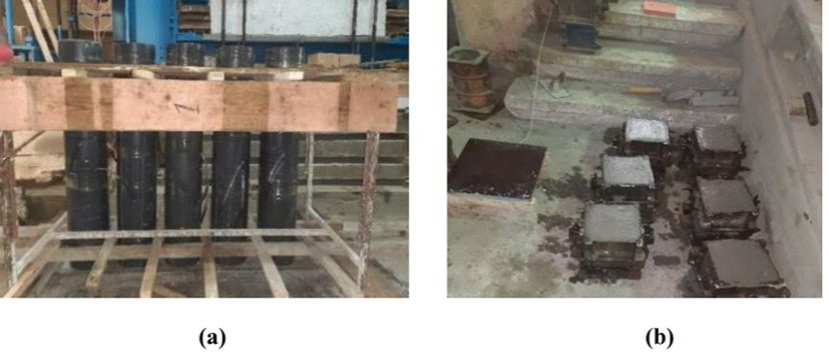
Specimens’ moulds (**a**) Columns (**b**) Cubes.

After the longitudinal and transverse reinforcements were assembled for each specimen, the steel cage was placed inside a PVC pipe with a diameter of 200 mm (shown in Fig. 3a). Next, for each mix, cement, sand, and coarse aggregate (NCA and/or RCA) were dry-mixed for 2 min. Then, 2/3 of the required water was added, and mixing continued for another 2 min. Finally, the remaining water and water reducer admixtures were added, and mixing continued for an additional 3 min before the specimens were cast. Once mixing was completed, the slump test was conducted to assess the workability, after which the mix was poured into the formwork. It is worth noting that the The column formwork was positioned on a rigid, well-leveled base to ensure a perfectly horizontal lower surface. Additionally, the upper surface of each column was carefully leveled during casting to achieve parallel end faces. These measures ensured concentric loading, prevented specimen tilting, and provided uniform contact with the loading platens, thereby minimizing the risk of eccentric loading.

Six cubes, each with dimensions of 150 mm × 150 mm × 150 mm (shown in Fig. 3b), were cast from each mix to test its compressive strength. The columns and cubes were cured by spraying water and covering them in a semi-controlled environment at 19–21 °C and 35–45% relative humidity for 28 days.

### Test setup and procedure

**Fig. 4:**
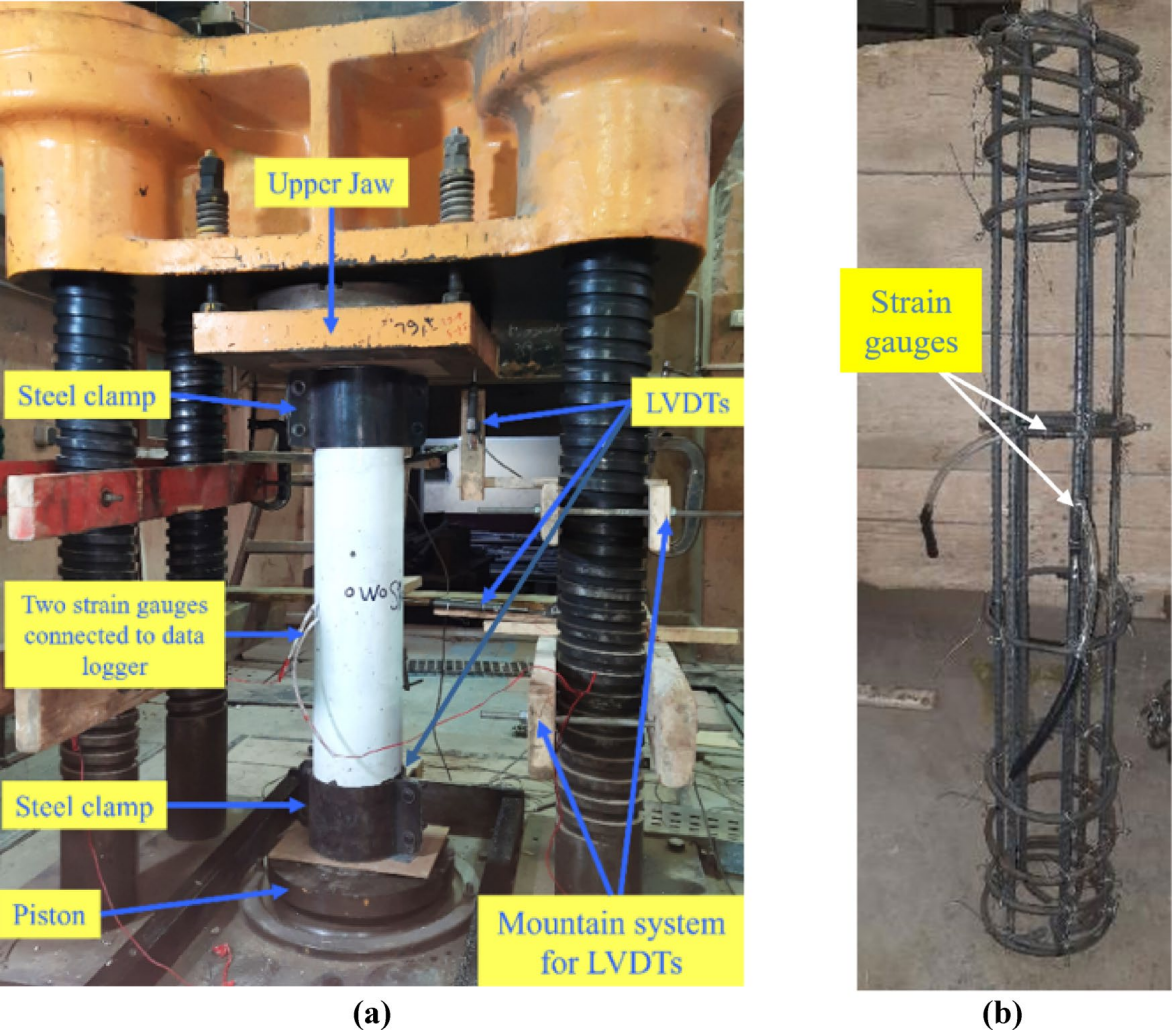
(**a**) Compression testing machine and column setup. (**b**) Location of strain gauges.

Prior to testing, each specimen was centered on the loading platen using alignment markings to align its longitudinal axis with the machine’s load axis, ensuring concentric loading and preventing eccentric stresses. Additionally, steel clamps (with a height of 180 mm) and rubber pads were used to ensure uniform load application and prevent bearing failure at the column’s upper and lower ends. The load was then applied axially using a 5000 kN capacity compression testing machine (see Fig. 4a) at a constant displacement rate of 0.5 mm/min until failure occurred. During loading, two linear variable differential transformers (LVDTs) measured lateral displacement at the column’s mid-height from two perpendicular sides, while two additional LVDTs monitored vertical displacement. Two strain gauges were also used to measure strain—one on the first stirrup near the column’s mid-height and the other at mid-height on the longitudinal bars (Fig. 4b). Loading was stopped once a significant decrease in load and excessive damage to the concrete cover was observed.

## Results and discussion

### Compressive strength of concrete mixes

**Fig. 5:**
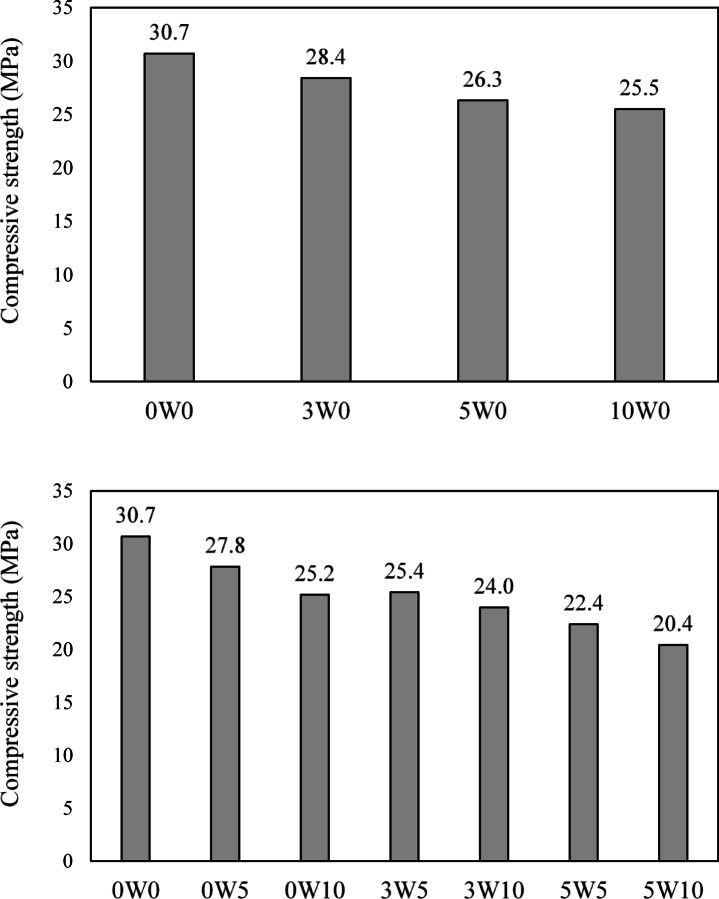
Compressive strength of all developed mixes.

Figure 5 shows the compressive strength of all developed mixes. As seen, the control mix developed with NCA and PW (0W0) exhibited a compressive strength of 30 MPa. As the RCA replacement increased, the compressive strength decreased. Mixes with 30%, 50%, and 100% RCA exhibited compressive strengths that were 7.5%, 14.3%, and 16.9% lower, respectively, than the control mix. These results align with findings by Abera^15^, where compressive strength reductions of 3.3%, 6.25%, and 19% were observed for mixes containing 30%, 50%, and 100% RCA, respectively. Similarly, Bahat^14^ observed a decrease of 13% and 21% for mixes with 50% and 100% RCA, respectively. The decrease in compressive strength may be due to the increased ITZs, which are generally the weakest areas in concrete composites. Along with the existing ITZ between the original aggregate particles and the old mortar, a new ITZ forms between the new mortar and the RCA particles. Additionally, the mechanical crushing process used to produce RCA can lead to damage and cracking within the mortar of RCA particles, as well as weaken the bond in the ITZ between the old mortar and the original aggregate particles. These factors collectively result in a reduction in the concrete’s overall strength.

The inclusion of TWW in concrete mixes also reduced the compressive strength of concrete. Compared to 0W0, the compressive strength decreased by 9.3% and 18.0% for mixes with NCA and TWW replacement levels of 50% and 100% (0W5 and 0W10), respectively. These reductions could be attributed to the presence of dissolved salts, sulfates, and chlorides in the TWW, which can interfere with cement hydration and weaken the cementitious matrix. Additionally, organic matter and residual chemicals may delay setting and reduce the bond strength between the cement paste and aggregates. The presence of suspended solids can also alter the effective water-to-cement ratio and increase porosity, further contributing to the observed strength loss. This finding is consistent with the results reported by Yao et al.^12^, Hassani et al.^13^, and Morgado et al.^14^. The combination of both RCA and TWW resulted in greater reductions in concrete strength. The mixes 3W5, 3W10, 5W5, and 5W10 showed strength reductions of 17.2%, 21.9%, 27%, and 33.4%, respectively, when compared to 0W0. Although RCA and/or TWW decayed the compressive strength of concrete, the failure pattern of all specimens was comparable with no significant differences.

### Cracking and failure mode of columns

**Fig. 6:**
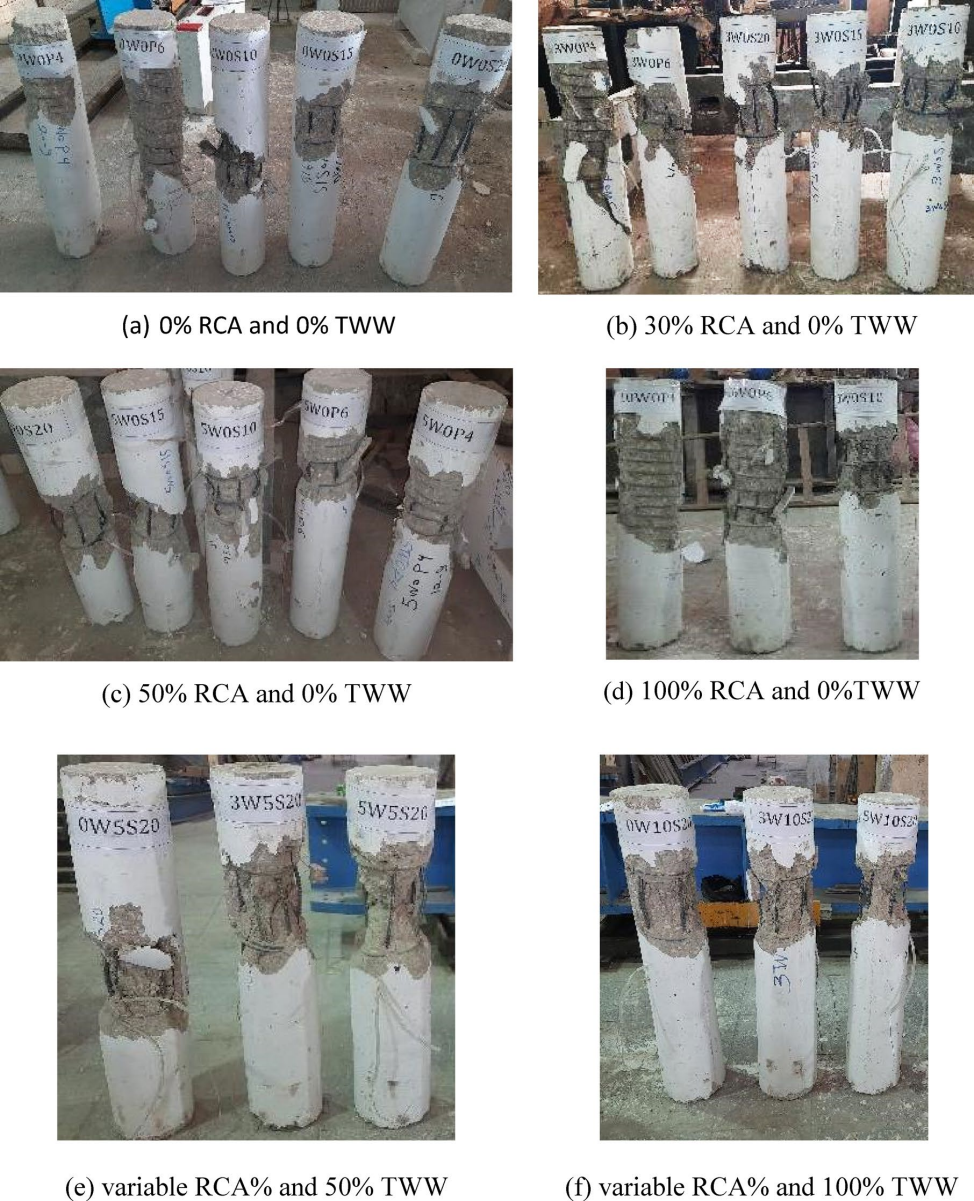
Crack pattern at failure of all tested columns.

Figure 6 shows the crack patterns of all tested columns at failure. In general, columns with discrete circular stirrups provided less uniform confinement. Their failure typically initiated with spalling of the concrete cover near the middle third of the column, followed by crushing of the inner concrete core and localized buckling of the longitudinal reinforcement. The extent of longitudinal bar buckling decreased as the stirrup spacing was reduced, indicating the influence of transverse reinforcement ratio on columns’ stability. In contrast, spiral columns consisted of a continuous helical bar wrapped around the longitudinal bars along the column height, providing uniform and continuous confinement. This continuous lateral restraint more effectively delays core crushing. Although spalling of the concrete cover still occurred, no significant buckling of the longitudinal reinforcement was observed. In all columns, vertical cracks were noted at the upper and lower one-third of the column height, particularly near the steel clamps at the column ends, likely due to stress concentrations. It is also worth noting that columns containing RCA and/or TWW showed greater internal damage. This increased deterioration is attributed to the combined effects of reduced concrete strength and altered microstructure caused by the use of RCA and TWW, which intensified cracking and core crushing compared to the control columns (with no RCA or TWW).

### Load–displacement behavior

**Fig. 7:**
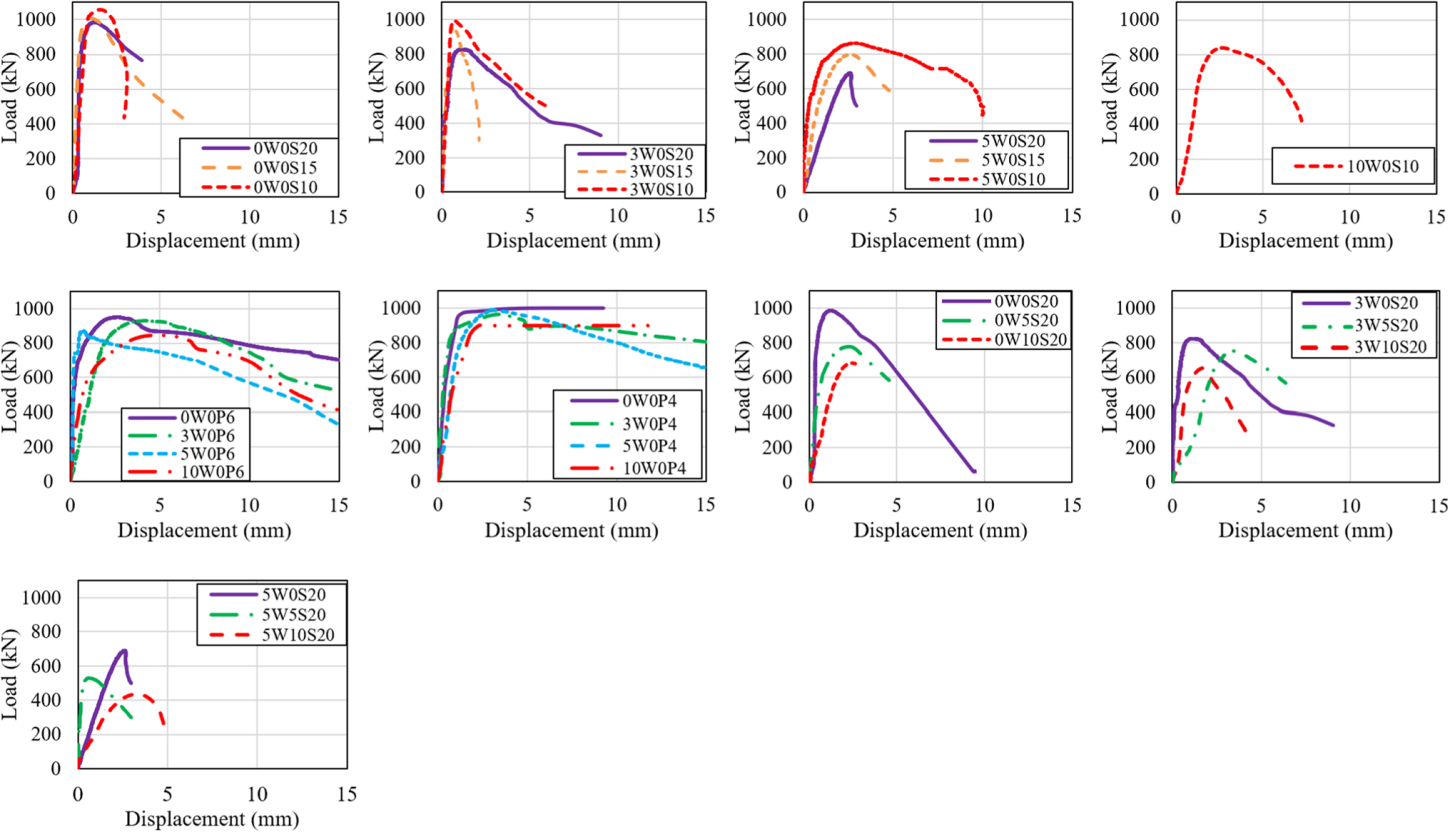
Load–displacement curves of all tested columns.

The load–displacement curves of all tested columns are shown in Fig. 7. It can be observed that the displacements at maximum load for all columns were relatively small, as the specimens were short columns, which typically do not experience significant displacements. The displacements at failure load ranged between 0.6 mm and 5.0 mm for all columns. From the figure, it can be seen that columns made with PW and RCA percentages of 0% and 30% exhibited nearly identical stiffness, likely because the relatively small proportion of RCA did not significantly alter the overall mechanical properties of the concrete, which is supported by the compressive strength results showing that the inclusion of 30% RCA caused only a 7.5% reduction. However, increasing the RCA to 50% and 100%, which caused greater reductions in concrete strength, allowed the concrete to deform more under axial load, leading to a corresponding decrease in column stiffness. As RCA content increased, the deformation corresponding to the ultimate load also increased; with RCA percentages of 0%, 30%, 50%, and 100%, the average column displacements at failure rose by 0%, 28.0%, 53.1%, and 106.6%, respectively. Similarly, introducing TWW into concrete led a reduction in columns stiffness and increased the deformability. For example, columns with 100% TWW demonstrated larger displacements at failure, with increases of 24.9% to 244.1% compared to columns made with PW. The lower stiffness and larger displacements observed in columns with RCA or TWW can be attributed to the negative effects of these materials in the microstructure or concrete, which typically facilitate crack propagation and result in greater deformation during loading. Figure 7 illustrates the effect of confinement provided by stirrups and spiral reinforcement. The inclusion of a tighter transverse reinforcement (i.e., reducing the spacing of stirrups or spiral pitch) generally mitigated the negative effects of RCA or TWW in most columns, resulting in higher stiffness. This is because the reinforcement confines the concrete core, limits lateral expansion, and enhances the axial load-carrying capacity, collectively increasing the apparent stiffness of the column.

However, compared to stirrup-reinforced columns, spiral reinforcement resulted in a larger area under the load–displacement curves and improved post-peak behavior, indicating higher ductility and energy absorption. Unlike stirrups, which provide local confinement at regular intervals, spiral reinforcement offers uniform and continuous confinement along the column height, allowing columns to sustain high loads, undergo larger deformations prior to failure, and exhibit a more gradual post-failure decay, reflecting increased safety.

### Column capacity

**Fig. 8:**
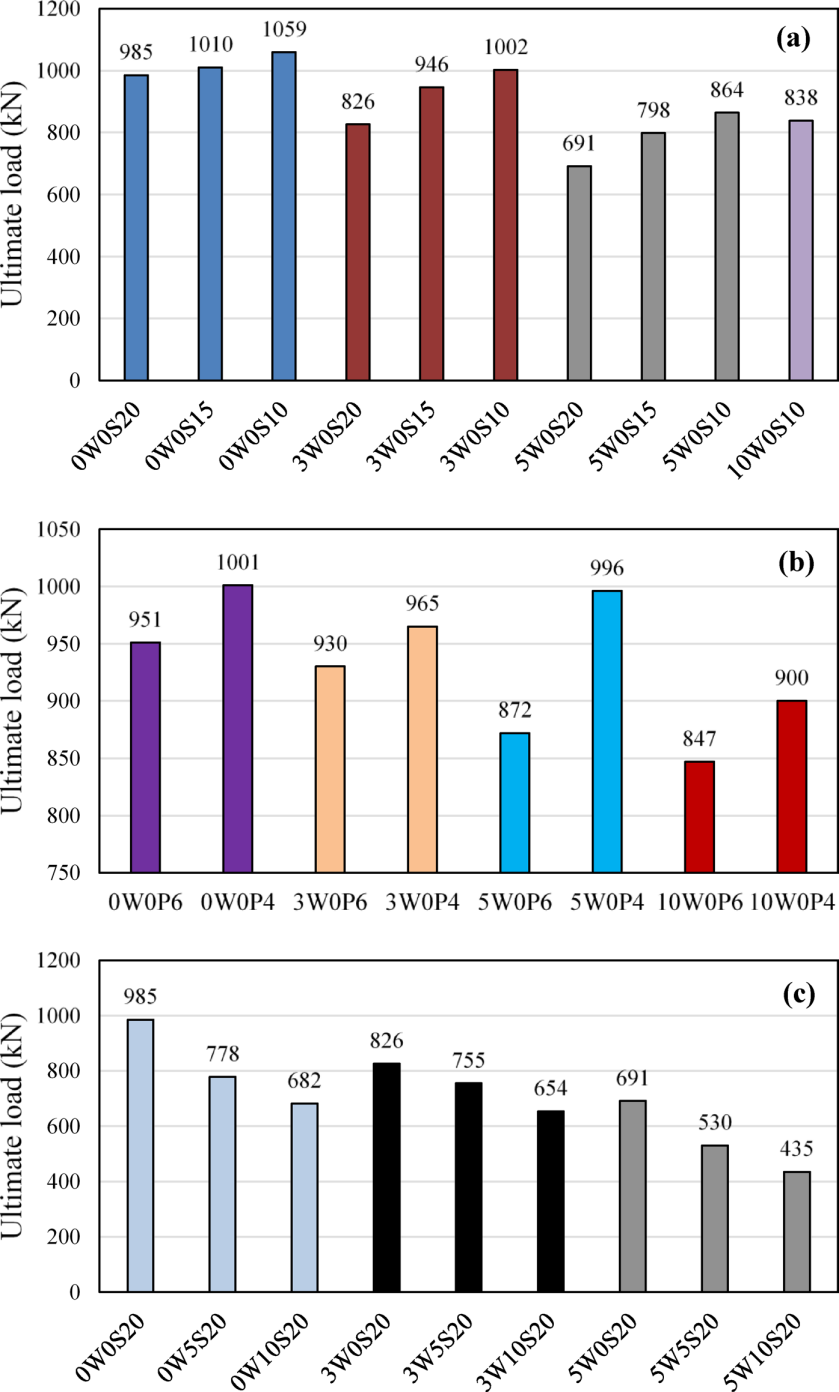
Failure load of the tested columns (**a**) stirrups-reinforced columns, (**b**) spirally reinforced columns, (**c**) columns with both RCA and TWW.

Figure 8 shows the experimental failure load of the test columns. From Fig. 8a, it is evident that the inclusion of RCA in the concrete generally led to a reduction in the ultimate capacity of the columns. Specifically, as the RCA replacement increased from 0 to 30% and 50%, the failure loads decreased by 16.1% and 29.8%, respectively, for columns with 200 mm spaced stirrups. In columns with 150 mm spaced stirrups, the failure loads decreased by 6.3% and 21%, respectively. Similarly, for columns with 100 mm spaced stirrups, the reductions in failure load were 5.4%, 18.4%, and 20.9% when 30%, 50%, and 100%, respectively.

For spiral columns (Fig. 8b), the reduction in failure load due to RCA replacement was notably smaller. The reductions in failure load were 2.2%, 8.3%, and 10.9% for columns with a 60 mm pitch, and 3.6%, 0.5%, and 10.1% for columns with a 40 mm pitch. These results suggest that the reduction in failure load due to increasing RCA replacement was less pronounced for spiral columns. This can likely be attributed to the higher confinement provided by the spiral reinforcement, which helps mitigate the negative effects of RCA inclusion. In contrast, columns with tied stirrups, which offer less confinement, showed more significant reductions in ultimate capacity as the RCA replacement level increased.

Figure 8a further demonstrates that the negative impact of RCA inclusion on column capacity can be mitigated by using closely spaced stirrups or a lower pitch spiral reinforcement. This suggests that confinement plays a crucial role in improving the structural performance of columns with RCA, counteracting the reduction in capacity caused by the use of recycled aggregates. For instance, at 30% RCA replacement and stirrups with a spacing of 150 mm, the column achieved 96% of the capacity of the reference column 0W0S20 (which had no RCA replacement). This indicates that while there was some reduction in capacity due to the RCA, the confinement provided by the 150 mm spaced stirrups was able to alleviate much of the negative effect. When the stirrup spacing was reduced to 100 mm, the confinement was significantly enhanced, fully compensating for the 30% RCA replacement and even surpassing the reduction in capacity caused by the RCA. In fact, columns with 100 mm spaced stirrups exhibited a performance that not only compensated for the 30% RCA but also provided additional strength, allowing the columns to exceed the original performance of columns with no RCA replacement. Reducing the stirrups space to 100 mm also allowed for up to 100% RCA replacement with a maximum capacity reduction of around 14.9%. Additionally, when considering higher RCA replacements, such as 50% and 100%, columns with 100 mm spaced stirrups reached 87.7% and 85.1% of the capacity of the 0W0S20 column, respectively. This highlights the beneficial role of closely spaced stirrups in mitigating the capacity loss due to higher RCA content.

Although, as shown in Fig. 8b, reducing the spiral pitch from 60 to 40 mm resulted in a slight increase in load-carrying capacity ranging from 3.7% to 6.3% across all RCA replacements, the use of spiral reinforcement, compared to discrete stirrups, provided a substantial compensation for the negative effects of RCA. At 30% RCA, columns with a spiral pitch of 60 and 40 mm achieved 94.4% to 98% of the capacity of the reference column (0W0S20), while columns with 50% RCA and a 60 mm spiral pitch reached 88.5% of the reference column’s capacity. It should be noted that the column with 50% RCA and a 40 mm spiral pitch exhibited a load-carrying capacity slightly higher than the reference column by 4.7%; however, this is considered a result of testing variability and is treated as an outlier. For columns with 100% RCA replacement, the load-carrying capacity reached 86% and 91.4% of the 0W0S20 reference column when spiral pitches of 60 mm and 40 mm were used, respectively, corresponding to reductions of 14% and 8.6%. These results further demonstrate that a lower pitch spiral reinforcement provides enhanced confinement, significantly reducing the detrimental effects of higher RCA content, and allowing for improved performance despite the increased proportion of recycled aggregates.

Figure 8c shows that as TWW content increased, the load-carrying capacity of the columns decreased. In columns cast with NCA, increasing the TWW replacement level to 50% and 100% led to a 21% and 30.7% decrease in failure loads, respectively, compared to the column with PW (0W0S20). For columns with 30% RCA, the reductions slightly increased to 23.4% and 33.6%, respectively. However, when both 50% RCA and TWW were incorporated, the reductions were more pronounced, reaching 46.2% and 55.8% for 50% and 100% TWW, respectively. These reductions are attributed to the combined negative effects of impurities in TWW and weakness of RCA, which hinder concrete strength development, as shown in the compressive strength results, ultimately reducing the columns’ overall load-bearing capacity.

### Comparison with code provisions

According to the ECP 203^41^ code, the failure load of columns can be obtained using the following equations (Eqs. 1, 2, 3, and 4):

- Stirrups-reinforced columns:

1 $${\text{P}}_{{\text{u}}} { = 0}{\text{.67 }}f_{cu} { }\left( {{\text{A}}_{{\text{c}}} - {\text{ A}}_{{{\text{st}}}} } \right){ + }f_{y} {\text{ A}}_{{{\text{st}}}}$$

- Spiral columns, (smaller of Eqs. 2 and 3):

2 $${\text{P}}_{{\text{u}}} { = 1}{\text{.14 }}\left( {{0}{\text{.67 }}f_{cu} \left( {{\text{A}}_{{\text{c}}} - {\text{A}}_{{{\text{st}}}} } \right){ + } f_{y} {\text{ A}}_{{{\text{st}}}} } \right)$$

3 $${\text{P}}_{{\text{u}}} { = 0}{\text{.67 }}f_{cu} { }\left( {{\text{A}}_{{\text{k}}} - {\text{A}}_{{{\text{st}}}} } \right){ + }f_{y} {\text{ A}}_{{{\text{st}}}} { + 2 }f_{yp } {\text{V}}_{{{\text{sp}}}}$$

4 $${\text{V}}_{{{\text{sp}}}} { = }\frac{{{\pi A}_{{{\text{sp}}}} {\text{ D}}_{{\text{k}}} }}{{\text{p}}}$$

where: ${\text{P}}_{{\text{u}}}$ is the ultimate load, ${\text{f}}_{{{\text{cu}}}}$ is the cube compressive strength of concrete at 28 days, ${\text{f}}_{{\text{y}}}$ is the longitudinal steel yield strength, ${\text{f}}_{{{\text{yp}}}}$ is the spiral stirrup steel yield strength, ${\text{A}}_{{\text{c}}}$ is the area of column section, ${\text{A}}_{{\text{k}}}$ is the area of concrete core enclosed by centerline of spiral stirrups, ${\text{A}}_{{{\text{st}}}}$ is the area of longitudinal steel bars, ${\text{V}}_{{{\text{sp}}}}$ is the spiral reinforcement ratio, ${\text{A}}_{{{\text{sp}}}}$ is the area of spiral stirrup, ${\text{D}}_{{\text{k}}}$ is the diameter of concrete core enclosed by centerline of spiral stirrup, and ${\text{p}}$ is the pitch of spiral stirrup, respectively.

According to the ACI 318^48^, the failure load of columns can be obtained using Eqs. 5 and 6:

- Stirrups-reinforced columns:

5 $${\text{P}}_{{\text{u}}} { = 0}{\text{.80 [0}}{.85 }f_{c}^{ ^{\prime}} { }\left( {{\text{A}}_{{\text{g}}} - {\text{A}}_{{{\text{st}}}} } \right){ + }f_{y} {\text{ A}}_{{{\text{st}}}} ]$$

- Spiral column:

6 $${\text{P}}_{{\text{u}}} { = 0}{\text{.85 [0}}{.85 }f_{c}^{ ^{\prime}} { }\left( {{\text{A}}_{{\text{g}}} - {\text{A}}_{{{\text{st}}}} } \right){ + }f_{y} {\text{A}}_{{{\text{st}}}} ]$$

where: ${\text{f}}_{{\text{c}}}{\prime}$ is the characteristic strength of the concrete calculated by multiplying the cube compressive strength by 0.8.to obtain the equivalent compressive strength of cylinder,.

According to the CSA A23.3^49^ code, the failure load of columns can be obtained using the following equations (Eqs. 7, 8, and 9):

- Stirrups-reinforced columns:

7 $${\text{P}}_{{\text{u}}} { = 0}{\text{.80 }}\left[ {{\upalpha }_{{1}} f_{c}^{ ^{\prime}} { }\left( {{\text{A}}_{{\text{g}}} - {\text{A}}_{{{\text{st}}}} } \right){ + }f_{y} {\text{ A}}_{{{\text{st}}}} } \right]$$

- Spiral column:

8 $${\text{P}}_{{\text{u}}} { = 0}{\text{.85 }}\left[ {{\upalpha }_{{1}} f_{c}^{ ^{\prime}} { }\left( {{\text{A}}_{{\text{g}}} - {\text{A}}_{{{\text{st}}}} } \right){ + }f_{y} {\text{A}}_{{{\text{st}}}} } \right]$$

9 $${\upalpha }_{{1}} { = 0}{\text{.85}} - {0}{\text{.0015 }}f_{c}^{ ^{\prime}} { } \ge { 0}{\text{.67}}$$

where: ${\upalpha }_{1}$ is the ratio of average stress in rectangular compression block to the specified concrete strength.

**Fig. 9:**
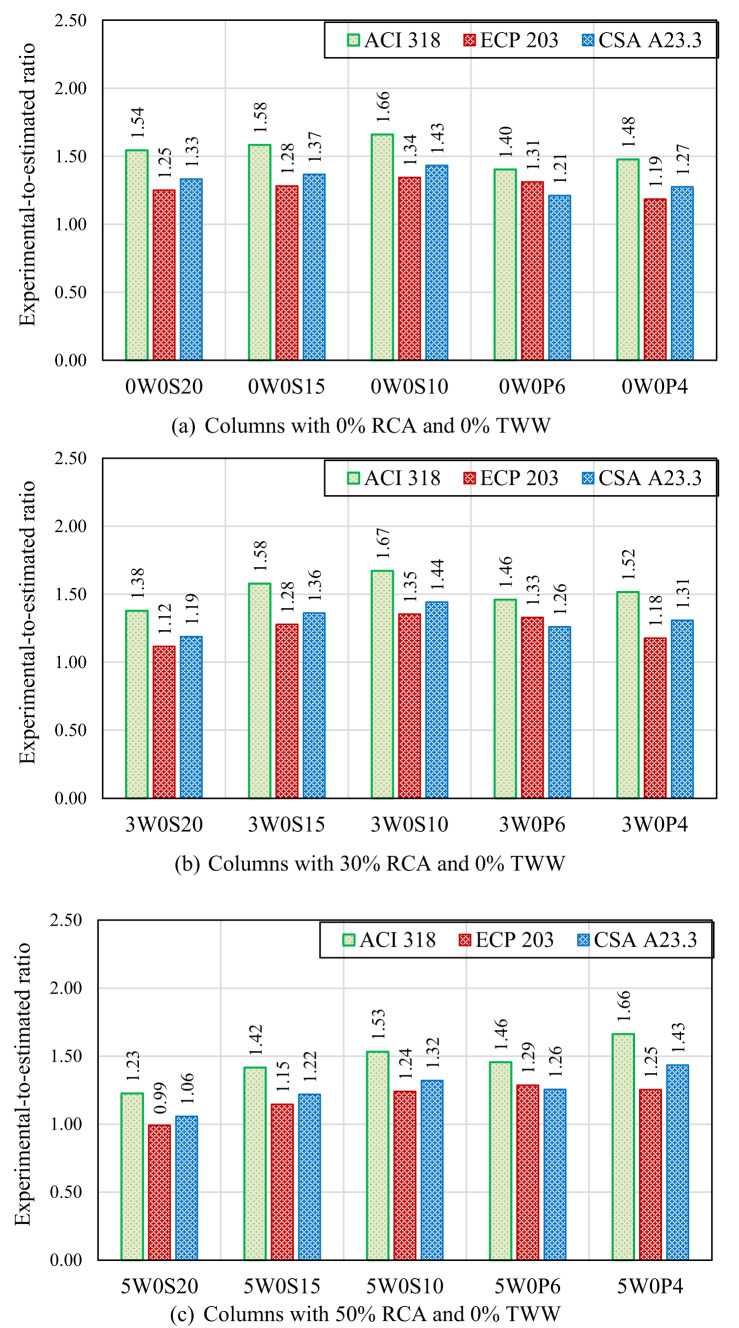

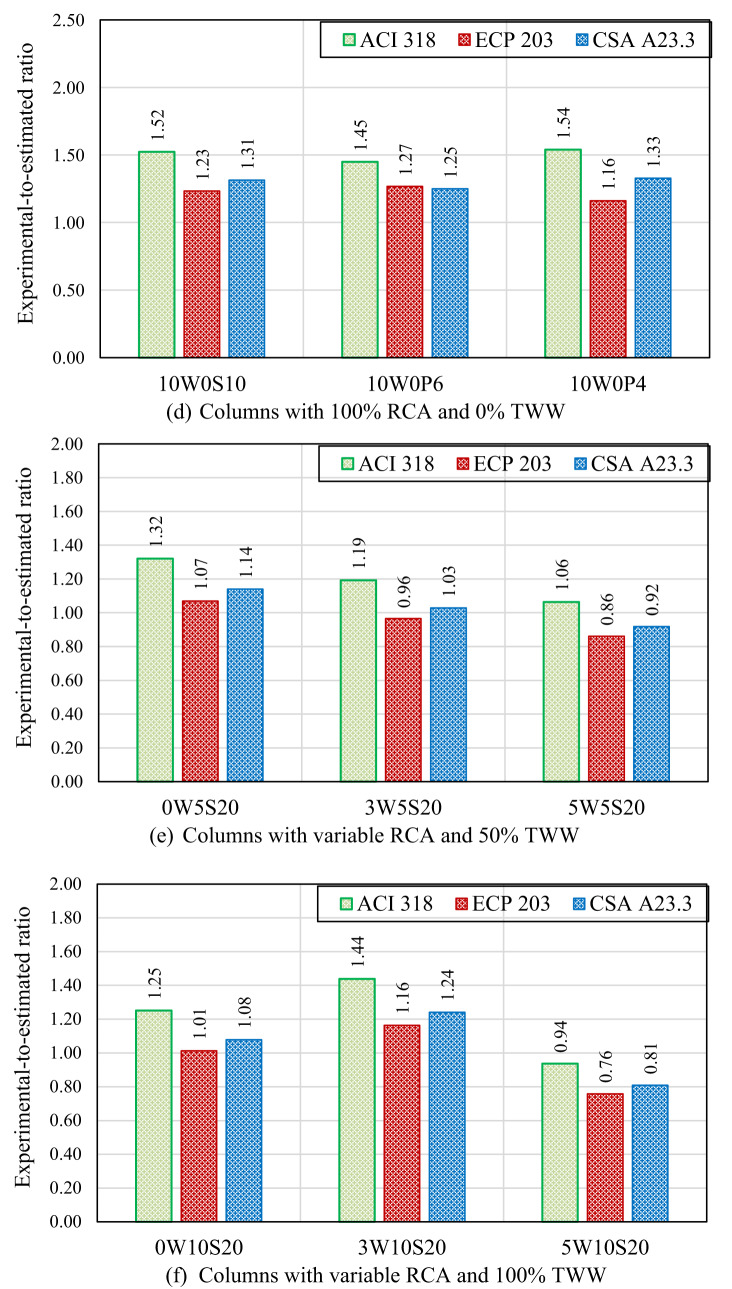
Comparison between experimental results and code provisions.

Figure 9 presents the ratio of the experimental failure load to the values estimated by the ACI 318, CSA A23.3, and ECP 203 codes for each tested column. The comparison revealed that the experimental failure loads of the columns cast without RCA or TWW exceeded the values estimated by all three design codes for both stirrups and spiral reinforced columns. For columns with stirrups, the ratios of experimental-to-estimated loads ranged from 1.54 to 1.66 for ACI 318, from 1.25 to 1.34 for ECP 203 (the lowest conservative among the codes), and from 1.33 to 1.43 for CSA A23.3, demonstrating that all codes provided conservative predictions. The use of 30% RCA had no noticeable effect on these estimations, as the experimental-to-estimated load ratios remained similar to those observed for specimens without RCA. However, beyond 30% RCA, load estimations became less conservative because the existing code equations, developed for natural aggregates, did not account for the negative effects of recycled aggregate on concrete strength. These effects—including additional weak zones at the interfaces between old and new materials, damage to RCA particles and old mortar from the crushing process, and higher porosity—are not fully captured in the codes and consequently led to lower ultimate loads than those predicted. As shown, at 50% and 100% RCA replacements, the experimental-to-estimated failure load ratios were decreased, ranging from 1.23 to 1.53 for ACI 318, 0.99 to 1.24 for ECP 203, and 1.06 to 1.32 for CSA A23.3. In spiral columns, those without RCA exhibited experimental-to-estimated failure load ratios ranging from 1.40 to 1.48 for ACI 318, 1.19 to 1.31 for ECP 203, and 1.21 to 1.27 for CSA A23.3. At 30% RCA replacement, similar ratios were observed compared with the corresponding columns without RCA (0W0P6 and 0W0P4 compared with 3W0P6 and 3W0P4, respectively). At 50% RCA replacement, the influence of spiral reinforcement became more pronounced, improving the margin of safety and resulting in experimental-to-estimated failure load ratios ranging from 1.46 to 1.66 for ACI 318, 1.25 to 1.29 for ECP 203, and 1.26 to 1.43 for CSA A23.3. This demonstrates that the confinement provided by the spiral reinforcement enabled the columns to sustain higher loads than those predicted by code equations, which are based on the reduced concrete strength associated with RCA. In contrast, at 100% RCA replacement, the safety margin decreased compared with columns containing 50% RCA contents, indicating that the negative impact of full RCA replacement outweighed the beneficial effect of spiral confinement on the experimental capacity. However, as a general observation from Fig. 9, for both stirrups and spiral reinforced columns, decreasing the stirrup spacing or spiral pitch resulted in an increased margin of safety. This improvement is attributed to the confinement effect of closely spaced transverse reinforcement, which enhances the column capacity but is not explicitly accounted for in the code equations.

Columns containing TWW, either alone or combined with RCA, showed poorer estimations, with experimental failure loads that were close to or lower than the values predicted by the three codes. For columns 0W5S20, 3W5S0, 5W5S20, 0W10S20, and 3W10S20, the experimental-to-estimated failure load ratios ranged from 0.86 to 1.44 across all three codes. Column 5W10S20 exhibited the least conservative predictions, with experimental-to-estimated ratios of 0.94, 0.76, and 0.81 for ACI 318, ECP 203, and CSA A23.3, respectively. Although code equations account for nominal concrete strength, the use of TWW can inherently reduce actual concrete performance in ways not captured by average concrete compressive strength alone, due to impurities, altered water–cement interactions, and increased variability—effects that are not fully reflected in the codes. When combined with RCA, these effects are compounded, further lowering the actual column capacity compared with code predictions. Such results highlight the need for modifications in the design code equations to better account for the negative impact of RCA and TWW, ensuring more reliable and realistic strength estimations.

## Conclusions

This study aimed to evaluate the effect of incorporating recycled coarse aggregate (RCA) and treated wastewater (TWW) on concrete behavior both at the material and structural levels, by testing reinforced concrete (RC) circular columns. Ten different mixtures were developed, with RCA incorporated at replacement levels of 0%, 30%, 50%, and 100%, and TWW used at replacement levels of 0%, 50%, and 100%. A total of 24 RC circular columns were cast from these mixes, each reinforced with various types of transverse reinforcement—stirrups at spacings of 200 mm, 150 mm, and 100 mm, as well as spiral reinforcement at pitches of 60 mm and 40 mm. All columns were tested under uniaxial loading. The ultimate capacity, cracking behavior, and load–displacement characteristics of the tested columns were analyzed and compared. Based on the findings, the following conclusions were drawn:

1. Increasing RCA content led to a reduction in compressive strength, with mixes containing 30%, 50%, and 100% RCA showing strength losses of 7.5%, 14.3%, and 16.9%, respectively, compared to the control mix (0W0). The addition of TWW also decreased strength, with reductions of 9.3% and 18.0% for 50% and 100% TWW replacements. The combination of RCA and TWW resulted in greater strength losses, with reductions ranging from 17.2% to 33.4%, demonstrating that each material adversely affects concrete strength and that their combined use further amplifies this reduction.
2. The replacement of RCA and TWW did not significantly alter the crack patterns or failure modes of the columns. However, their combined use led to increased internal damage, particularly in the columns with insufficient transverse reinforcement (i.e., spacing 200 mm). This highlights the critical role of transverse reinforcement, in maintaining the structural integrity and performance of concrete columns.
3. With the incorporation of RCA and/or TWW, the stiffness of the columns decreased, resulting in larger displacements. However, reducing stirrup spacing or using spiral reinforcement improved stiffness and reduced deformability. Spiral columns, in particular, exhibited larger deformations due to enhanced confinement.
4. Increasing RCA content reduced the failure load of columns, with reductions of 16.1% and 29.8% for 30% and 50% RCA replacement in columns with 200 mm spaced stirrups. Spiral columns showed smaller reductions, with 2.2% to 10.9% loss in failure load due to better confinement. Closely spaced stirrups (100 mm) allowed for up to 30% RCA replacement with no reduction in capacity, and up to 100% RCA replacement with a maximum capacity reduction of around 14.9%. Similarly, using spiral reinforcement with a 40 mm pitch enabled up to 100% RCA replacement with only an 8.6% reduction in capacity, compared to columns made with conventional concrete.
5. Increasing TWW content also decreased load capacity, with reductions of 21% and 30.7% for 50% and 100% TWW in columns with NCA. When RCA and TWW were used together, the reductions became more pronounced, reaching up to 55.8% for columns with 50% RCA and 100% TWW, demonstrating their detrimental effect on the columns’ load-carrying capacity.
6. The comparison between experimental results and code predictions indicates that all three design codes (ACI 318, CSA A23.3, and ECP 203) provide conservative estimates of column capacity, particularly for mixes without RCA or with up to 30% RCA replacement. Beyond 30% RCA, the codes become less conservative. Columns incorporating TWW, or a combination of RCA and TWW, exhibited experimental loads that were close to or lower than the code estimates, indicating that the current codes’ equations do not fully capture the influence of RCA or TWW. This highlights the need for code revisions to better account for the effects of these alternative materials. Moreover, the use of tighter transverse reinforcement spacing resulted in an increased margin of safety, attributed to the enhanced confinement provided by closely spaced transverse reinforcement—an effect not explicitly considered in the code equations.

## Limitations and future directions

While this study provides valuable insights into the effects of RCA and TWW on the strength and behavior of RC circular columns, several limitations should be acknowledged. First, the experimental program was limited to a relatively small number of mixes and column specimens, which may restrict the generalizability of the findings to all structural configurations, concrete grades, or environmental conditions. Second, only monotonic concentric loading was considered, and the performance under cyclic, eccentric, or dynamic loads was not investigated, which are common in real structural applications. Third, the study focused on short-term strength and load-carrying capacity, without considering long-term durability, shrinkage, creep, or corrosion effects associated with RCA and TWW. Additionally, the study was limited to specific levels of RCA and TWW replacement and specific reinforcement arrangements, which may not cover the full range of practical applications.

Future research should address these limitations by expanding the experimental program to include a wider variety of column sizes, reinforcement types, and loading conditions. Investigations into long-term durability, environmental exposure effects, and serviceability performance are needed to ensure safe and sustainable use of RCA and TWW in concrete structures. Moreover, developing predictive models and updating design codes to account for the combined effects of RCA, TWW, and reinforcement detailing would enhance the practical applicability of sustainable concrete in structural design.

## Data Availability

All data generated or analyzed during this study are included in this published article.
